# 

**Published:** 1896-03

**Authors:** 


### CONTENTS.

ORIGINAL ARTICLES:
Notes on Veterinary Education and Intelligence, Concerning Veterinary and Agricultural Colleges. By W. B. NILES, D.V.M. . . . 169
How Shall the Practitioner Continue His Education? By DR. R. A. ARCHIBALD 174
Report on Southern Cattle Fever. By W. H. HARBAUGH, V.S. . . . 177
Health of the Pig. By D. MCINTOSH, V.S. . . . 184
The Theories of Immunity. By E. P. NILES, D.V.M. . . . 189
Veterinary Meat-inspection. By G. ED. LEACH, M.D.C. . . . 194
Purpura Hemorrhagica in the Bovine Species, with Report of a Case. By G. T. NETHERTON, M.D.C.. . . . 199
ABSTRACTS FROM FOREIGN JOURNALS:
American Horses for the French Army—Strangles in South Africa—Malignant Œdema—Malaria in Animals—Compulsory Meat-inspection and Cattle-insurance in Saxony—The Treatment of Shoulder-boils or Cold Abscess of the Shoulder—Death of Count Taaffe . . . 201–210
PERSONAL . . . 210
REPORTS OF CASES:
An Unpleasant Though Interesting Experience. By JNO. E. BROWN, V.S. . . . 211
Case of Equine Rabies. By B. M. UNDERHILL, V.M.D. . . . 214
Fracture of Radius. By DR. L. M. KLUTTS . . . 215
Experiments with Barium Chloride. By J. J. MCCARREY . . . 218
Ligation of the Carotid Artery. By E. H. MORRIS . . . 220
CORRESPONDENCE . . . 222
CONTROL WORK . . . 224
EDITORIAL:
The College Chart for 1896 . . . 227
Legislation in 1896 . . . 228
MARRIED—NECROLOGY . . . 231
SOCIETY PROCEEDINGS:
Abstract of Stenographer's Report, U. S. V. M. A.—Montreal Veterinary Medical Association—Virginia State Veterinary Medical Association—Maine Veterinary Medical Association—Veterinary Medical Association of New York County—Michigan State Veterinary Medical Association—New Hampshire Veterinary Medical Association—Keystone Veterinary Medical Association—The Ohio State Veterinary Medical Association—Pennsylvania State Veterinary Examining Board . . . 232–250
AT YOUR LEISURE . . . 251
NEW PUBLICATIONS . . . 252

